# Highlight: Ancient Human Polymorphisms Linked to Modern-Day Health Concerns for Native Americans

**DOI:** 10.1093/gbe/evz105

**Published:** 2019-05-31

**Authors:** Casey McGrath

Long-chain polyunsaturated fatty acids (PUFAs) are essential for human brain development and immunity. Although they can be obtained by consuming certain foods, especially seafood, the largest dietary source of PUFAs is plant oils. However, plant-derived PUFAs are shorter and must be converted into longer chains by enzymes known as fatty acid desaturases (FADS). Research over the last decade has revealed that humans vary in the efficiency of their FADS enzymes, and this can be attributed in large part to variation in the genes encoding these enzymes. In a new study in the current issue of *Genome Biology and Evolution* entitled “Evolution of hominin polyunsaturated fatty acid metabolism: from Africa to the New World,” a group of researchers led by Timothy O’Connor at the University of Maryland provides an in-depth look at the history of these genes in human populations, with particular relevance for the health of present-day Native Americans (Harris et al. 2019).

The ancestor of all humans had two *FADS* genes similar to those found in nonhuman primates, termed the ancestral haplogroup. Most individuals of African ancestry, however, have *FADS* genes that encode more efficient enzymes, termed the derived haplogroup, whereas populations from Europe, East Asia, South Asia, and Oceania have a mix of both haplogroups. Based on previous research with a relatively small sample, it appeared that Native Americans primarily carried the ancestral haplogroup. The presence and origin of the ancestral haplogroup, which encodes less efficient FADS enzymes, in non-African populations have long puzzled researchers. Explanations have ranged from differences in environmental selective pressures to interbreeding between modern humans and Neanderthals or Denisovans, archaic hominins known to carry the ancestral haplogroup. In their new study, O’Connor et al. used data collected from almost 3,000 individuals as part of the 1000 Genomes Project, GeneSTAR, and the Peruvian Genome Project to shed light on this mystery.

In their analysis, the authors confirmed that >99% of Native Americans carried the ancestral haplogroup, a finding that could have health and nutritional consequences for individuals of Native American ancestry. According to first author Daniel Harris, currently a PhD candidate working with O’Connor, this is the most important result of the study from a health perspective. “The ancestral haplogroup is associated with lower long-chain PUFA levels,” notes Harris. “Since [these] are essential elements of human physiology, this poses a potential public nutrition problem for individuals with Native American ancestry, especially in light of the modern Western diet.” In other words, depending on their dietary intake of foods containing short- and long-chain PUFAs, it may be more difficult for those of Native American ancestry to produce sufficient amounts of long-chain PUFAs for optimal brain development and immunity.

The authors then turned to explaining the high prevalence of the ancestral haplogroup in the Native American population. The researchers found no evidence that these haplogroup patterns are due to interbreeding with Neanderthals or Denisovans. Instead, it may be that the ancestral versions of the *FADS* genes are somehow linked to adaptation to cold weather or the dietary restrictions of a cold climate. Interestingly, in Siberia, O’Connor et al. demonstrated that the ancestral haplogroup was more common in more northern regions, supporting this theory. The authors also found evidence for positive selection on the *FADS* genes in Native Americans. According to Harris, however, this issue for now remains unresolved: “We need a better picture of the ancestral haplogroup’s frequency in the Native American founding population.” Harris notes that “this is essential for differentiating between positive selection of the ancestral haplogroup shortly after the Out-of-Africa expansion [and] recent positive selection in Native Americans.” This could be accomplished, says Harris, by increasing access to ancient DNA samples from East Asia and Siberia. In addition, Harris notes the need for studies investigating long-chain PUFA levels, as well as the potential health impacts of less efficient FADS enzymes, in Native American communities.
